# Rotavirus Vaccine Trials in International Centre for Diarrhoeal Disease Research, Bangladesh (icddr,b) and Future Use of the Vaccine in Bangladesh

**DOI:** 10.1093/infdis/jiab442

**Published:** 2021-09-16

**Authors:** K Zaman, Asma Binte Aziz, Md Yunus, Firdausi Qadri, Allen G Ross, John D Clemens

**Affiliations:** 1 International Centre for Diarrhoeal Disease Research Bangladesh, Dhaka, Bangladesh; 2 International Vaccine Institute (IVI), Seoul, Republic of Korea; 3 UCLA Fielding School of Public Health, Los Angeles, California, USA

**Keywords:** Bangladesh, efficacy study, rotavirus vaccine

## Abstract

Safe and effective rotavirus vaccines (RVs) are needed to reduce the enormous public health burden of rotavirus illness in developing countries. Vaccination is critical for effective control of rotavirus infection since it cannot be prevented with improvements in water and sanitation. The International Centre for Diarrhoeal Disease Research, Bangladesh (icddr,b) has completed several groundbreaking RV trials (Phase I–Phase IV). The safety, immunogenicity, efficacy, and effectiveness of different RVs were evaluated among both urban and rural populations. In this study, we present the results, policy implications, and lessons learned for successful implementation of these trials as well as future directions for rotavirus vaccination in Bangladesh.

Almost all infants without vaccination experience at least 1 rotavirus infection by 2 years of age, and approximately 118 000 children under 5 years of age died globally due to rotavirus in 2019 [1]. Most deaths occur in low- and middle-income countries, especially in Africa and Asia [2]. Many of the deaths can be prevented if children had access to safe and effective rotavirus vaccines (RVs) where urgent care for severe rotavirus illness is often limited or even inaccessible. For decades the International Centre for Diarrhoeal Disease Research, Bangladesh (icddr,b) played a significant role in evaluating RV safety, immunogenicity, efficacy, and effectiveness in high-mortality, low-income settings in Bangladesh. These vaccine studies, led by the icddr,b, made an important contribution to the growing body of evidence that demonstrated the safety, efficacy, and potential lifesaving impact of RVs, and they have accelerated the availability of rotavirus vaccine through the recommendation of the World Health Organization (WHO) since June 2009. Currently, 110 countries have already introduced the rotavirus vaccine in their national immunization programs [3]. However, the battle against one of the leading childhood killers is yet to be over because more than 40 percent of the world’s children still do not have access to the rotavirus vaccine. Many children with no access to the rotavirus vaccine live in low-income settings of Asia and Africa, which delays the introduction of the vaccine in routine immunization programs. In addition, due to the high price of the rotavirus vaccines (approximately US $19–24 per dose) and lack of adequate cold chain facilities, many countries cannot introduce the vaccine in the routine immunization program. Furthermore, low-income or slum-dwelling people cannot afford, and they are the ones who need it the most. There are 3 million infants in the annual birth cohort without access to rotavirus vaccines in Bangladesh where 1 diarrheal disease hospitalization can cost more than 20% of the average monthly household income for an extremely poor family [3, 4]. Bangladesh can save an estimated US $66.8 million annually (Jahangir Hossain, verbal communication, 20 January 2020) by introducing the rotavirus vaccine in routine immunization programs, which will prevent approximately 135 000 hospitalizations per year.

## THE icddr,b MODEL

The icddr,b is the world’s largest diarrheal hospital and treats more than 200 000 patients a year. For the last 50 years, its research continues to evaluate low-cost, scalable vaccines against rotavirus, cholera, other enteric and respiratory diseases and different noninterventional public health approaches. It is notable that its development of oral rehydration solution has saved tens of millions of lives and created a profound impact on global health crises. With the support of highly knowledgeable and skilled scientists, clinicians, researchers, nurses, health workers, and auxiliary staff with unparallel experience, icddr,b can undertake various investigations on emerging infectious diseases and new interventions to reduce maternal and neonatal mortality.

The icddr,b has conducted several RV trials ranging from Phase I to III and postmarketing evaluation of the vaccines (Phase IV). Three US Food and Drug Administration-licensed rotavirus vaccines were tested and evaluated in the icddr,b trials: tetravalent rhesus rotavirus vaccine ([RRV-TV] RotaShield; Wyeth Lederle), pentavalent rotavirus vaccine ([PRV] RotaTeq; Merck & Co.), and monovalent rotavirus vaccine (Rotarix; GlaxoSmithKline) [2, 5–7]. We also studied the coadministration of rotavirus vaccines with oral polio vaccine (OPV) provided through routine immunization programs [6]. These trials were conducted in both urban and rural areas, and the trial sample sizes ranged from a few hundred to several thousand. The safety, immunogenicity, efficacy, and effectiveness of the vaccines were evaluated (Table 1).

**Table 1.** Rotavirus Vaccine Studies Conducted by icddr,b

Vaccine Candidate	Study Year	Study Type and Design	Vaccine Schedule	Age of Administration	Study Groups	No. of Participants	Results
RRV-TV [5]	1998–1999	Phase II, randomized, double-blinded, placebo-controlled trial	3 doses	6, 10, and 14 weeks	RRV-TV	55	87% Immunogenic
					Placebo	54	32% Immunogenic
Human monovalent vaccine (Rotarix) [6] (ClinicalTrials.gov NCT00139334)	2005–2006	Phase II, randomized, double-blinded, placebo-controlled trial	2 doses	12 and16 weeks	Rotarix +OPV	100	57% Immunogenic
					Rotarix	100	67% Immunogenic
					Placebo	100	19% Immunogenic
RotaTeq [7] (ClinicalTrials.gov NCT00362648)	2007–2009	Phase III, randomized, double- blinded, placebo-controlled trial	3 doses	6, 10, and 14 weeks	RotaTeq	1018	48% Efficacious
					Placebo	1018	
Human monovalent vaccine (Rotarix) [2] (ClinicalTrials.gov NCT 00737503)	2008–2011	Phase IV, open-label, cluster-randomized trial	2 doses	6 and 10 weeks	Rotarix	6527	41% Effective
					OPV	5791	
Heat-stable rotavirus vaccine (Hilleman Laboratories and RotaTeq) [8] (ClinicalTrials.gov NCT02728869)	2016–2017	Phase I/II, randomized controlled trial	3 doses	6, 10, and 14 weeks	Heat- stable rotavirus vaccine	25	88% Immunogenic
					RotaTeq	25	84% Immunogenic

Abbreviations: icddr,b, International Centre for Diarrhoeal Disease Research, Bangladesh; OPV, oral polio vaccine; RRV-TV, rhesus rotavirus tetravalent vaccine.

## TRIAL OUTCOMES

The first rotavirus vaccine trial conducted by icddr,b was a Phase II randomized, double-blinded, placebo-controlled trial to test the safety and immunogenicity of RRV-TV during the period of 1998–1999 in rural Bangladesh. The study showed that RRV-TV was comparably immunogenic (87% seroresponse rate) and safe as other trials conducted in the developing world [5]. The study data confirmed that rural children with common malnutrition can have immunologic responses to vaccination like those in developed countries and supported future trials of rotavirus vaccines in developing countries [5]. We conducted a Phase I study with human monovalent vaccine (Rotarix) among 90 toddlers in 2002 to determine the safety of the vaccine in an urban slum area (Zaman et al. 2002, unpublished). Another Phase II randomized, placebo-controlled immunogenicity study was conducted during 2005–2006 among 300 healthy infants living in an urban slum to test the safety and immunogenicity of 2 doses of the human monovalent vaccine (Rotarix) when coadministered with OPV, which is routinely administered at 6, 10, and 14 weeks of age. The study findings strongly suggested that Rotarix can be concomitantly given with OPV in routine immunization without interference in immunogenicity and facilitated the decision on rapid and effective integration of rotavirus vaccine into the routine Expanded Programme on Immunization (EPI) in many countries [6].

The icddr,b conducted a Phase III clinical study among 2036 infants to assess the safety, immunogenicity, and efficacy of the 3-dose RotaTeq vaccine administered at 6, 10, and 14 weeks of age along with Vietnam and scientists from other institutions who simultaneously conducted trials in 3 sites in Africa (Kenya, Ghana, and Mali). The study showed a significant reduction of severe rotavirus disease by 51% in the first year of life, when children are at the greatest risk of diarrhea-related illness and death. This finding supports expansion of the WHO recommendations to promote the global use of the rotavirus vaccine [7]. Our study was the first clinical efficacy trial of an already licensed rotavirus vaccine in developing countries in Asia which showed that even with lower efficacy than the data reported in trials of RotaTeq in developed countries, rotavirus vaccine can significantly reduce the burden of severe disease [7]. We conducted the first effectiveness study in a cluster-randomized design of a rotavirus vaccination program using Rotarix in rural Bangladesh, where rotavirus diarrhea rates were compared between clusters with and without Rotarix, allowing the first examination of overall, direct, and indirect protection by a rotavirus vaccine. The study showed that 2 doses of Rotarix vaccine works well under programmatic conditions and is 41% effective against severe acute rotavirus diarrhea, although indirect protection of nonvaccinees was not demonstrable [2]. This was the first study to provide real-world efficacy data on the WHO-recommended, 2-dose rotavirus vaccine schedule in a low-resource setting in Asia. The study provided valuable insight on research to understand programmatic means to address waning effectiveness after vaccination and the need for booster doses to improve and extend protection [2]. We also compared a new heat-stable formulation of lyophilized live-attenuated pentavalent rotavirus vaccine (HSRV)— manufactured by MSD Wellcome Trust Hilleman Laboratories (New Delhi, India) with RotaTeq—in a randomized Phase I/II among infants living in urban slum, which was the first trial of its kind. The HSRV was found to be safe and immunogenic (88% seroresponse rate) [8]. This heat-stable rotavirus vaccine can be sustained at 45°C for 7 months, and this is important for resource-limited settings where most of the rotavirus burden exists and maintaining a cold chain is challenging. Our study generates new data on newer rotavirus vaccine formulations that can partially or entirely eliminate cold chain dependence and reduce associated costs [8].

## FUTURE DIRECTIONS

Our experience with rotavirus vaccine trials indicates that that all phases of clinical trials with good clinical practice standards can be conducted maintaining high quality and coverage in urban slums and in rural populations of Bangladesh. For all trials, we were able to enroll a large number of study participants within the timeline and foster high compliance of study-related activities. We conducted these studies in vulnerable slum and rural populations by maintaining a community relationship, which benefitted the study population to a large extent. The rotavirus-effectiveness study provided experience with the vaccine in a “real-world” setting, and valuable lessons were learned on routine vaccine delivery in Bangladesh.

A few challenges were faced in the trials, such as maintaining proper temperature of vaccines in very hot weather and carrying vaccine cold boxes during rainy season with difficult transportation system, but it was managed successfully because of very careful forward planning. There were no unacceptable temperature deviations recorded during vaccine and sample storage or transportation, nor any record of vaccine vial damage. Retention of study participants in a mobile population, particularly in urban areas with migration rates of approximately 25% per year, was hard to achieve. The study clinic provided outpatient services for the study population 7 days a week from 8:30 am to 5:00 pm and remained open for 24 hours for any emergency services. After office hours, mothers could easily communicate with study medical officers and respective field staff over the phone to ensure necessary treatment of their infants. Standard medical care was provided free of cost, and we referred patients to the nearby hospitals when inpatient services were needed. All these initiatives highly encouraged the community in both the urban slums and rural Bangladesh to take part in our studies over many years.

In 2016, Bangladesh applied to Gavi for support for rotavirus vaccination, and they expected to introduce Rotarix in the routine immunization in 2018 [9]. Gavi-funded support covers the cost of rotavirus vaccine procurement and a one-time grant to cover introduction costs in eligible countries. This allowed countries in Africa and Asia, where most rotavirus deaths occur, to introduce the vaccine. However, the introduction in Bangladesh was delayed due to lack of infrastructure and the need to expand and upgrade the cold chain facilities to support the planned EPI expansion in the whole country [10]. There was also an escalation in the target population for all vaccinees from 3.25 million to 3.76 million during the proposed time frame. Although the focus was first on the peripheral cold storage expansion, which was mostly complete, central storage facility timelines were significantly pushed back. Furthermore, time, resources, and funding were limited for rotavirus vaccine introduction due to competing priorities with the measles-rubella vaccination campaign planned in late 2019 [11], to overcome the burden of measles in the country that began to surge around the time Gavi approved Bangladesh’s application for rotavirus vaccination. Overall, insufficient readiness of Bangladesh EPI and lack of planning to guide a realistic introduction timeline contributed to the substantial delay for the rotavirus vaccine introduction in Bangladesh, and introduction has not begun at the time of this writing.

We are optimistic that Bangladesh will hasten the process to introduce the rotavirus vaccine initially approved in principle. We need strong political commitment and approval from different national immunization committees (scientific and technical subcommittees) to ensure that appropriate attention and energy is focused on implementing the country’s rotavirus vaccination plans. Furthermore, the decision to introduce any new vaccines also depends on the different studies conducted by research organizations, particularly on disease burden and cost-effectiveness analysis. Coordination among EPI officials, global and national rotavirus experts, vaccine developers, and concerned people is crucial to explain the intervention to the policymakers to convince them to move forward and take the necessary action. Even after the introduction of the rotavirus vaccine, there can be a shortage of supply; hence, there must be a backup plan for possible alternative products for uninterrupted vaccine supply. A coalition with neighboring India, which contains the developers of 2 recent WHO prequalified rotavirus vaccines (ROTAVAC [Bharat Biotech] and ROTASIIL [Serum Institute of India]), can make the transition easier. Soon after a vaccine is introduced, postvaccination surveillance is important to identify effectiveness, manage postvaccination risk (eg, intussusception) to understand the impact of vaccination, and undertake postvaccination control measures [12]. The routine introduction of rotavirus vaccine will ultimately save lives and bring hope for a brighter future.

## Notes

***Acknowledgments.*** The International Centre for Diarrhoeal Disease Research, Bangladesh (icddr,b) is grateful to the Governments of Bangladesh, Canada, Sweden, and the United Kingdom for providing core support.

***Potential conflicts of interest.*** All authors: No reported conflicts of interest. All authors have submitted the ICMJE Form for Disclosure of Potential Conflicts of Interest.

## References

[1] Global Burden of Disease Collaborative Network. Global Burden of Disease Study 2019 (GBD 2019) Results. Institute for Health Metrics and Evaluation (IHME). Available at: http://ghdx.healthdata.org/gbd-results-tool. Accessed 25 August 2021.

[2] Zaman K, Sack DA, Neuzil KM, et al. Effectiveness of a live oral human rotavirus vaccine after programmatic introduction in Bangladesh: a cluster-randomized trial. PLoS Med 2017; 14:e1002282. 28419095 10.1371/journal.pmed.1002282 PMC5395158

[3] International Vaccine Access Center, Vaccine Information and Epidemiology Window (VIEW-hub). RV Current Vaccine Intro Status. Available at: http://www.view-hub.org/. Accessed 15 April 2021.

[4] Sarker AR, Sultana M, Mahumud RA, et al. Economic costs of hospitalized diarrheal disease in Bangladesh: a societal perspective. Glob Health Res Policy 2018; 3:1. 29318195 10.1186/s41256-017-0056-5 PMC5755417

[5] Bresee JS, El Arifeen S, Azim T, et al. Safety and immunogenicity of tetravalent rhesus-based rotavirus vaccine in Bangladesh. Pediatr Infect Dis J 2001; 20:1136–43. 11740320 10.1097/00006454-200112000-00009

[6] Zaman K, Sack DA, Yunus M, et al.; Bangladeshi Rotavirus Vaccine study group. Successful co-administration of a human rotavirus and oral poliovirus vaccines in Bangladeshi infants in a 2-dose schedule at 12 and 16 weeks of age. Vaccine 2009; 27:1333–9. 19162114 10.1016/j.vaccine.2008.12.059

[7] Zaman K, Dang DA, Victor JC, et al. Efficacy of pentavalent rotavirus vaccine against severe rotavirus gastroenteritis in infants in developing countries in Asia: a randomised, double-blind, placebo-controlled trial. Lancet 2010; 376:615–23. 20692031 10.1016/S0140-6736(10)60755-6

[8] Kanchan V, Zaman K, Aziz AB, et al. A randomized Phase I/II study to evaluate safety and reactogenicity of a heat-stable rotavirus vaccine in healthy adults followed by evaluation of the safety, reactogenicity, and immunogenicity in infants. Hum Vaccin Immunother 2020; 16:693–702. 31526218 10.1080/21645515.2019.1664239 PMC7227685

[9] Pecenka C, Parashar U, Tate JE, et al. Impact and cost-effectiveness of rotavirus vaccination in Bangladesh. Vaccine 2017; 35:3982–7. 28623028 10.1016/j.vaccine.2017.05.087 PMC5512265

[10] World Health Organization. The Effective Vaccine Management (EVM). Available at: https://www.who.int/immunization/programmes_systems/supply_chain/evm/en/. Accessed 15 April 2021.

[11] World Health Organization. Global Measles and Rubella Update: April 2018. Available at: https://www.who.int/immunization/monitoring_surveillance/burden/vpd/surveillance_type/active/Global_MR_Update_April_2018.pdf.

[12] Aziz AB, Ali M, Basunia AH, Yunus M, Clemens J, Zaman K. Impact of vaccination on the risk factors for acute rotavirus diarrhea: an analysis of the data of a cluster randomized trial conducted in a rural area of Bangladesh. Vaccine 2020; 38:2190–7. 31983585 10.1016/j.vaccine.2020.01.041

